# Genotype VII.1.1-Based Newcastle Disease Virus Vaccines Afford Better Protection against Field Isolates in Commercial Broiler Chickens

**DOI:** 10.3390/ani12131696

**Published:** 2022-06-30

**Authors:** Abdelmonem A. A. Dewidar, Walid H. Kilany, Azza A. El-Sawah, Salama A. S. Shany, Al-Hussien M. Dahshan, Islam Hisham, Magdy F. Elkady, Ahmed Ali

**Affiliations:** 1 Poultry Diseases Department, Faculty of Veterinary Medicine, Beni-Suef University, Beni-Suef 62511, Egypt; abdwidar@yahoo.com (A.A.A.D.); azzasawah@yahoo.com (A.A.E.-S.); salama.shany@vet.bsu.edu.eg (S.A.S.S.); hussiendahshan73@vet.bsu.edu.eg (A.-H.M.D.); mfelkady@vet.bsu.edu.eg (M.F.E.); 2 Reference Laboratory for Veterinary Quality Control on Poultry Production (RLQP), Agriculture Research Center, Animal Health Research Institute, Giza 12618, Egypt; walidhamdy78@yahoo.com (W.H.K.); islamhisham@vetmed.dmu.edu.eg (I.H.)

**Keywords:** Newcastle disease, genotype VII.1.1, VG/GA, recombinant vaccine, broiler chickens

## Abstract

**Simple Summary:**

Controlling genotype VII Newcastle disease virus (NDV) is challenging, especially in endemic countries. Genetic engineering was used to develop recombinant vaccines against NDV (rNDV). The close genetic relationship with circulating viruses can better protect against field NDV challenges. This study evaluated two commercial rNDV genotype VII.1.1 vaccines based on the LaSota strain backbone or VG/GA strain backbone compared to conventional genotype II vaccines. Both vaccines induced a protective immune response; however, GII-based vaccines failed to prevent virus shedding efficiently. Additionally, the noticeable superior performance of the rNDV vaccine based on the VG/GA strain backbone may be attributed to the enterotropic nature of the VG/GA strain, which makes it replicate more efficiently in both the respiratory and intestinal tracts of chickens. Future research needs to evaluate the cell-mediated immune response induced by the rNDV GVII vaccines to understand their mechanism better mediating the mucosal immunity.

**Abstract:**

This study evaluated the efficacy of live and inactivated conventional GII LaSota and recombinant GVII Newcastle disease vaccines in commercial broilers. The experimental groups (G2–G7) were vaccinated on day 7 and day 21 of age with live vaccines from the same vaccine type “GII LaSota, GVII vaccine (A), GVII vaccine (B)” via eye drop; however, G3, G5, and G7 received a single dose from inactivated counterpart vaccines subcutaneously on day 7 of age. Vaccine efficacy was evaluated based on elicited humoral immunity, clinical protection, and reduction in virus shedding after challenge with virulent GVII 1.1. strain. Results demonstrated that live and inactivated recombinant GVII vaccine based on VG/GA strain backbone elicited superior protection parameters (100% protection). Although the conventional GII LaSota live and inactivated vaccination regime protected 93.3% of vaccinated birds, the virus shedding continued until 10 DPC. The post-vaccination serological monitoring was consistent with protection results. The study concludes that conventional GII ND vaccines alone are probably insufficient due to the current epidemiology of the GVII 1.1 NDV strains. Our findings further support that protection induced by recombinant GVII 1.1. ND vaccines are superior. Interestingly, the efficacy of recombinant ND vaccines seemed to be influenced by the backbone virus since the VG/GA backbone-based vaccine provided better protection and reduced virus shedding.

## 1. Introduction

Newcastle disease (ND) caused by the Newcastle disease virus (NDV) is still one of the most devastating viral diseases of poultry worldwide. The clade VII.1.1 of the sub-genotype VII.1 of the class II NDV [1,2] is the most common clade causing several outbreaks in Africa, Asia, Europe, and South America despite extensive vaccinations using live and inactivated NDV vaccines with multiple regimes [3]. Most of the used vaccines belong to genotypes I and II NDV viruses that partially protect chickens from mortalities and clinical signs, but NDV shedding from infected birds to the environment persists [4].

The high genetic difference between the currently circulating genotype VII (GVII) field isolates and the genotype I or II vaccine strains was reported to play a critical role in the frequent exposure of poultry farms to virulent NDV outbreaks despite extensive vaccination strategies [5,6,7,8]. For instance, many strains of the GVII NDV showed a single-point mutation in the hemagglutinin-neuraminidase (HN) gene linear epitope (E347K) that allows evading the virus by its specific antibodies [9]. Moreover, serological and animal testing found distinguishable antigenic variation between NDV genotype VII and the commercial vaccine strain LaSota, as indicated by 2-fold and 3–6-fold differences in HI and neutralization titers, respectively [10].

Therefore, homologous genotype VII.1.1. NDV vaccines were proposed to provide a higher efficacy [11,12]. Though both heterologous and homologous NDV vaccines provide clinical protection, genetically matched GVII vaccines showed higher hemagglutination inhibition (HI) antibody titers and superior reduction in the virulent NDV shedding titers [13,14]. Besides being safe as live NDV vaccines, mutations in the large protein gene of the recombinant genotype VII.1.1 NDV (rNDV GVII) strains were found to induce a progressive elevation of the homologous antibodies and markedly higher CD8^+^ T cell percentage, T cell proliferation, and IFN-γ than LaSota strain vaccines [15].

Different types of rNDV GVII vaccines have been developed by incorporating the fusion (F) into different backbones viruses, including the NDV GII LaSota or Villegas-Glisson/University of Georgia (VG/GA) viruses or other viruses, e.g., herpesvirus of turkeys (HVT) virus [16]. The cloning of genotype VII.1.1 viruses F and HN genes into the NDV G II strains yields promising vaccine candidates that provide better clinical protection and reduce virus shedding than classical GII vaccines [17]. However, the potential alteration in protection acquired following a primary or booster vaccination by the newly developed rNDV GVII vaccines will need to be tested by using different vaccination regimens in commercial broilers in the presence of maternal antibodies [18]. Additionally, comparisons of the use of different NDV GII backbones in terms of protection levels against field viruses are also required.

Therefore, the current study was conducted to evaluate the efficacy of NDV commercially available GVII.1.1. live and killed vaccines in a prime-boost vaccination regime to protect broiler chickens against the challenge of a recent GVII.1.1 NDV strain. Along with biosecurity measures, our findings may present an additional recommended step in NDV control.

## 2. Materials and Methods

### 2.1. Viruses and Vaccines

#### 2.1.1. Vaccines

The used NDV vaccines in the study included GII Volvac^®^ LaSota gold (live attenuated) and Volvac^®^ AI + ND (killed vaccine) (Boehringer Ingelheim, Ingelheim am Rhein, Germany). Two rNDV GVII.1.1 vaccines were included in the study; the Himmvac Dalguban N (Plus) live vaccine/Himmvac Dalguban N (Plus) killed vaccines, abbreviated: vaccine (A) GVII” (KBNP, Inc., Anyang, Korea) and the live attenuated RINNOVAC™ ELI-7/MEVAC ND 7 Plus inactivated, abbreviated: vaccine (B) GVII” (MEVAC, Cairo, Egypt). The rNDV vaccine (A) is a chimeric recombinant GVII based on the LaSota strain backbone that contains the F and HN genes of the GVII 1.1. KBNP-C4152R2L strain [17]. However, the vaccine (B) is based on a VG/GA strain backbone into which the F and HN genes of the CK/ME/19 strain were inserted. Both vaccines A and B strains belong to the NDV GVII.1.1 (Supplementary Figure S1A); however, they share 94.5 and 96.4% nucleotide and amino acid identities, respectively. Compared to the classical NDV GII vaccine strains (i.e., LaSota and VG/GA strains), the KBNP-C4152R2L and CK/ME/19 strains of vaccines A and B share 83.4–84.4 and 88.1–89% nucleotide and amino acid identities, respectively (Supplementary Figure S1A).

#### 2.1.2. Diagnostic Antigens

NDV diagnostic antigens were prepared using the LaSota (Volvac® LaSota gold strain) and GVII 1.1 (vNDV strain 567F, acc. no: JX647839) strain for the hemagglutination inhibition (HI) test. Viruses were propagated via inoculation into the allantoic sac of specific pathogen-free embryonated chicken eggs (SPF-ECE ) incubated at 37 °C for 72–96 h. Inoculated eggs allantoic fluids from dead and surviving embryos were harvested after overnight chilling at 4 °C and tested for hemagglutination using 1% washed chicken red blood cells. The antigens had 8–9 log2 hemagglutinating units (HAU)/25 µL, respectively. Four hemagglutinating units (4HAU) were adjusted for the HI test. Back titration of antigen was carried out to verify the number of HAU used. The HI test was conducted according to the OIE Manual of Diagnostic Tests and Vaccines for Terrestrial Animals 2021 [19] using 4 HA units of each antigen, and two-fold dilution of the sera and titers were determined using 1% chicken red blood cells.

#### 2.1.3. Challenge Virus

The velogenic viscerotropic Newcastle disease virus (vNDV) [567F] strain was used to challenge the vaccinated birds and the control ones. The strain was isolated via inoculation of the allantoic sac of 10-day-old embryonated SPF chicken eggs (GenBank accession number: JX647839). The virus was propagated in SPF-ECE as described in Section 2.1.2. The virus Embryo Infective Dose 50 (EID50) was determined via titration in 10-day-old SPF-ECE according to the method described by Reed and Munech [20].

### 2.2. Evaluation of Different Vaccination Regimes (Genotype II- and Genotype VII-Based Vaccines) against NDV Field Isolate Challenges

#### 2.2.1. Chicken Experiments

One hundred and twenty mixed-sex commercial Cobb broiler chicks (day-old) with maternally derived antibodies (MDA) were randomly divided into eight groups of 15 birds each. The experimental birds were kept inside poultry raising rooms under controlled conditions for the study period and received regular feed and water *ad libitum*. All the experimental procedures were reviewed and approved by the Animal Care and Use Committee (ACUC), Faculty of Veterinary Medicine, Beni-Suef University (n° BSU/VetMed-2021/0303). The birds were vaccinated with either NDV genotype II or genotype VII-based vaccines according to the regimes shown in (Table 1). Blood samples were collected from the wing vein at 21 and 31 days to determine the NDV-specific antibody titers using the hemagglutination inhibition (HI) test. On day 31 of age, birds were challenged with 10^6^ EID_50_/0.1 mL of a challenge virus via the intranasal route. After the challenge, birds were monitored daily for clinical signs of disease (edema, muscular tremors, torticollis, and paralysis of wings and legs) and mortality. Chickens displaying severe clinical signs of disease were euthanized. Tracheal swabs were collected from randomly selected five birds at 3-, 7-, and 10- DPC to determine the virus shedding.

#### 2.2.2. Virus Shedding Titers Determination

To investigate the NDV shedding, 5 tracheal swabs were required assuming 99% expected NDV-positive swabs, ±5.0% precision, with a 95% confidence interval and 1 design effect [21]. However, swabs were collected from all birds in each group and pooled (1 pool = 3 swabs) in 2 mL of phosphate buffer saline (PBS) pH 7.0–7.4, clarified by centrifugation at 4000 rpm for 10 min, then processed for RNA extraction [22] using the QIAamp Viral RNA Mini Kit (Qiagen, Hilden, Germany) according to the manufacturer’s instructions. All samples were tested for the presence of RNA of challenge NDV strain using Ag Path real-time one-step RT-qPCR kit (Thermo Fisher Scientific, Waltham, MA, USA) using the F gene-specific primers to detect virulent NDV genotype VII.1.1 [23], as shown in Table 2.

#### 2.2.3. Tissues Histopathology

At 3 and 7 DPC, three chicks from each group were humanely euthanized, and organs (trachea and cecal tonsils) were collected. Tissue samples were fixed in 10% neutral-buffered formalin, embedded in paraffin, sectioned at 4μm, stained by hematoxylin and eosin (H&E), and examined by light microscopy [24]. For lesion scoring, a numerical scoring of tracheal tissues was used. Briefly, histopathological tracheal injuries were scored in degrees as follows: -: no change; +: <25% tissue damage; ++: 26–50% tissue damage; +++: 51–75% tissue damage; ++++: 76–100% tissue damage.

### 2.3. Statistical Analysis

Data on parameters of interest were systematically collected and analyzed using GraphPad Prism version 9.00 for Windows (GraphPad Software, San Diego, CA, USA, www.graphpad.com). The variations within groups using different antigens for the HI test and in-between groups were analyzed using two-way ANOVA, and significant differences were determined by the Sidak multiple comparisons test. A Student’s t-test was run between all groups after ANOVA to confirm the statistical significance. A *p*-value < 0.05 was considered statistically significant.

## 3. Results

### 3.1. Serological Response in Different Newcastle Disease Virus Vaccination Regimes

Generally, the unvaccinated group did not exhibit detectable specific antibodies neither at 21 nor 31 days of age using both LaSota GII and GVII 1.1 diagnostic antigens. The post-vaccination immune response evaluated using LaSota GII diagnostic antigen has demonstrated that G3: LaSota GII LI/L had the highest mean HI antibody titers (3.67 ± 1.0) on day 21 of age. On day 31 of age, G4: Vaccine (A) GVII L/L showed the lowest titers (2.89 ± 0.78). The remaining vaccinated groups did not show any significant differences from each other.

The results of serological testing of the same samples against GVII 1.1 diagnostic antigen showed that G7: Vaccine (B) GVII LI/L elicited superior post-vaccination immune response over the other vaccinated groups with mean HI antibody titers 4.5 ± 0.84 on day 21 of age. The antibody titers of the same group gradually developed thereafter with higher values (6.2 ± 1.03) on day 31 of age. Using different antigens for the same group revealed no significant difference except for the G7: Vaccine (B) GVII LI/L group, where titers measured by GVII 1.1 antigen were significantly higher (*p* < 0.05) than those measured by GII LaSota antigen at both ages (Table 3).

### 3.2. Clinical Protection of Different Live Attenuated and Killed Genotype VII.1.1-Based NDV Vaccines against NDV Virulent Challenge

**Figure 1:**
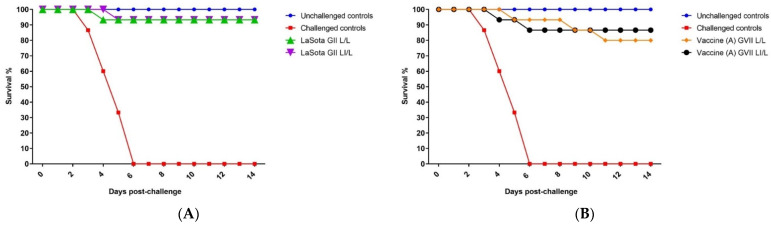

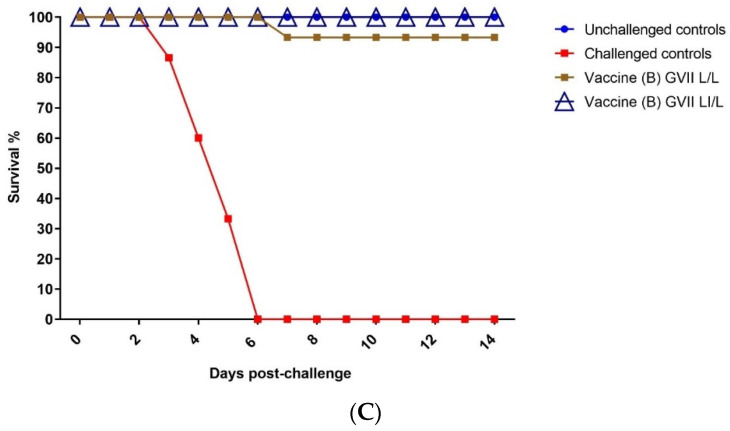
**Survival of vaccinated groups post challenge with virulent genotype VII.1.1. Newcastle Disease Virus.** (**A**) GII LaSota vaccinated group. (**B**) Vaccine (A) GVII vaccinated group. (**C**) Vaccine (B) GVII vaccinated group. Abbreviations; GII: genotype II-based vaccine, GVII: genotype VII.1.1.-based vaccine, L/L: live vaccine at 7 and 21 days, LI/L: live and inactivated at 7 days, then live vaccine at 21 days.

The validity of the vaccination challenge experiment was confirmed by the absence of any clinical signs and mortalities in the negative control group (G1). Meanwhile, the challenge control group (G8) exhibited typical vNDV clinical signs, including severe respiratory and nervous manifestations, and all birds died by 6 DPC (Figure 1). The results demonstrated that the protection level was 93.3% in LaSota vaccinated groups (G2 and G3), resembling L/L and LI/L regimes (Figure 1A). However, the estimated protection levels induced by the vaccine (A) GVII L/L and LI/L in G3 and G4 were 80% and 86.6%, respectively (Figure 1B). Meanwhile, the protection levels against the vNDV challenge were 93% and 100% in G6: vaccine (B) GVII LI/L and G7: vaccine (B) GVII L/L regime, respectively. (Figure 1C).

### 3.3. Challenge Virus Shedding

In all vaccinated groups, the reduction in virus shedding titers was significant at 3 DPC (1–1.5 log_10_ EID_50_/mL reduction, *p* ≤ 0.05); however, all the collected swabs were positive by RT-qPCR. The number of shedding birds was lower in most vaccinated groups by 7 DPC; 5/5 and 4/5 samples were positive for NDV shedding in the GII LaSota and vaccine (B) GVII L/L, respectively. In the remaining groups, only 2–3 out of 5 birds were shedding low virus titers (1.13 ± 0.16–1.94 ± 0.17 log_10_ EID_50_/mL). The virus shedding stopped entirely in the vaccine (A) LI/L and both vaccine (B) GVII L/L and LI/L groups by 10 DPC. Though the LaSota GII-based vaccines (G2 and G3) protected birds clinically, the virus shedding remained until 10 DPC in all tested tracheal swabs collected from challenged birds (Table 4).

### 3.4. Histopathology

**Figure 2:**
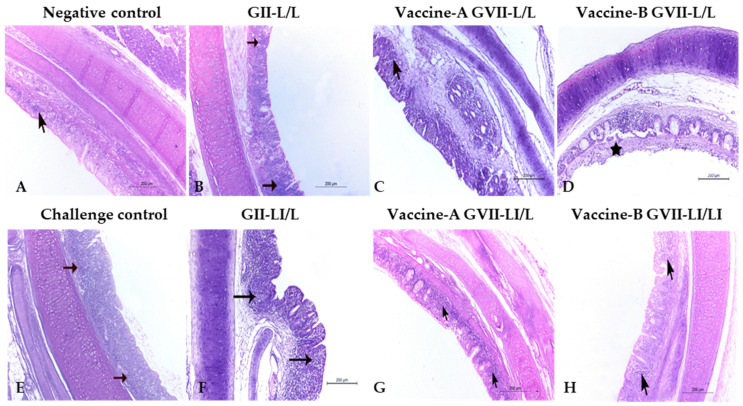
Trachea in vaccinated and control groups chicks at 3 days post challenge (H & E stain, ×100), showing the negative control normal histopathological architecture of the trachea (**A**). The challenge control (**E**). All vaccinated group’s (**D**,**H**) tracheal structures were as in the normal group except for the LaSota GII LI\L (**B**,**F**) and the vaccine-B GVII L/L (**C**,**G**) groups. Abbreviations: GII: genotype II-based vaccine, GVII: genotype VII.1.1.-based vaccine, L/L: live vaccine at 7 and 21 days, LI/L: live and inactivated at 7 days, then live vaccine at 21 days.

**Figure 3:**
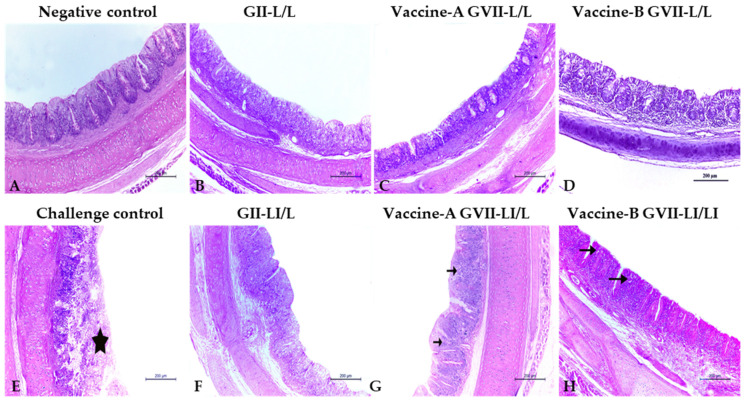
Trachea in vaccinated and control groups chicks at 7 days post challenge (H & E stain, ×100), showing the normal histopathological architecture of trachea with few lymphocytes in the submucosa and between mucosal epithelium in the negative control group (arrows) (**A**). The challenge control group showed severe deterioration of the tracheal structure and tracheal casts (star) (**E**). All vaccinated groups (**B**,**D**,**G**,**H**) had reduced tracheal mucosa damage, except for the vaccine-B GVII L/L (**C**) and the LaSota GII LI\L (**F**) groups. Abbreviations: GII: genotype II-based vaccine, GVII: genotype VII.1.1.-based vaccine, L/L: live vaccine at 7 and 21 days, LI/L: live and inactivated at 7 days, then live vaccine at 21 days.

The histopathology of the trachea and cecal tonsils collected from vaccinated groups showed that the live/inactivated ND vaccines at 7 days old followed by a live attenuated vaccine program is generally better than depending only on live vaccines at both time points. Collected organs histopathology at both 3 and 7 days post challenge showed relatively mild histopathological changes in all vaccinated groups (Figure 2 and Figure 3), except for the trachea of the vaccine (A) and (B) GVII L/L (Figure 2D) and the LaSota GII LI\L (Figure 2F) at 3 days post challenge that showed higher lesion scores of sloughing of the tracheal mucosa, congestion on the submucosal blood vessels, and eventually tracheal cast (Table 5). At 7 DPC, the trachea of the vaccine (A) GVII LI/L (Figure 3C), vaccine (B) GVII L/L (Figure 3D), and the LaSota GII LI\L (Figure 3F) showed higher scores of epithelial degeneration and submucosal lymphocytic infiltration (Table 5).

## 4. Discussion

ND remains the most serious avian disease with devastating effects on the poultry industry. The World Organization for Animal Health (OIE) has classified ND as a notifiable disease because of its global outbreaks and geographic dispersion [19]. ND is a highly infectious agent capable of causing high mortality in unvaccinated chickens and subclinical forms of ND in vaccinated and/or NDV-exposed flocks, which may act synergistically with other bacterial or viral infections, resulting in more severe disease and economic losses [25].

Although all NDV strains belong to a single serotype, they continually change, as do all other RNA viruses. Since the early 1990s, GVII.1.1 NDV strains have been associated with lethal infections of poultry flocks in Asia, Europe, and Africa [15]. Since ND has been involved in five pandemics worldwide since 1926, genotype VII.1.1 strains (former sub-genotypes VIIb, VIId, VIIe, VIIj, and VIIl) viruses were linked to the fourth panzootic [2].

Vaccination regimens against ND are more stringent, especially in endemic countries; however, outbreaks still occur [26]. Various factors, including the vaccine strain, the inhibitory effects of maternal antibodies, and the ability of live vaccines to induce stress and facilitate secondary infection contribute to the efficacy of vaccination programs [27]. Additionally, currently used genetically distant vaccines (i.e., GII-based vaccines, e.g., Hitchner B-1 and LaSota) do not effectively protect birds [28]. On the other hand, the co-infection with other pathogens (e.g., infectious bronchitis virus, infectious bursal disease virus, and Mycoplasma) might result in vaccine failure due to immunosuppression [29,30]. Moreover, the insufficient biosecurity measures and intensive vaccination probably exert a selection/vaccination pressure with multiple amino acid substitutions in the epitopes of NDV HN, which may affect the vaccine efficacy in preventing clinical disease and virus shedding [31].

Genotype-matched vaccines were found to offer superior protection against NDV infection [9,32,33]. Though inactivated GVII. 1.1-based vaccines protected vaccinated chickens with a significant decrease in the virus shedding titer compared to GII-based vaccines [6,34,35]. The need for a specific level of biosecurity for production hinders its wide use. The criteria for live vaccines, which are better inducers of local, humoral, and cell-mediated immunity, could not be achieved [34,36]. It is interesting to note that vaccination regimes implementing a prime-boost strategy using live and inactivated vaccines still need to be investigated and evaluated in the light of supporting the poultry industry in endemic areas. In this study, we evaluated the efficacy of NDV commercially available genotype-matched vaccines (i.e., GVII.1.1-based live and inactivated vaccines) in prime-boost-based programs against challenge with [567F] strain resembling circulating velogenic genotype VII.1.1 Newcastle Disease viruses.

Evaluation of NDV vaccines is not merely based on protection against clinical signs and mortality associated with the disease. The vaccine’s ability to minimize viral shed from vaccinated challenged birds is crucial for mitigating the spread of the disease [37]. Nevertheless, the level of protection is correlated with the HI antibody titers [38]. The amount shed will vary according to the host immunity, the quantity and virulence of the challenge virus, the dosage and kind of ND vaccine used, and the time interval between vaccination and challenge [39].

In the current study, testing circumstances were adjusted to simulate those encountered in the field. However, due to the requirements for working with vNDV, experimental birds were transferred before the challenge to isolators equipped with a HEPA filter and in a room with negative pressure and essential air exchanges. Commercial broiler chickens vaccinated with recombinant ND GVII vaccine (B) groups (G6 and G7) resembling live/live and live + inactivated/live models showed 93.3% and 100% protection against challenge with velogenic NDV genotype VII strain, respectively, without any notable clinical signs. Protection levels were 93.3% in groups vaccinated with conventional GII LaSota groups (G2 and G3). However, birds that received the recombinant GVII vaccine (A) in groups G4 and G5 showed 80% and 86.6% protection in live/live and live + inactivated/live models, respectively. None of the unvaccinated unchallenged control birds (G1) showed clinical signs or mortality associated with ND. Nevertheless, unvaccinated challenged control birds (G8) showed typical clinical signs of ND disease after the challenge, i.e., severe respiratory signs and neurological symptoms, and the group showed 100% mortality by the sixth day post challenge.

NDV HI antibody titers of 6 log_2_ or above after immunization protect chickens against clinical disease following virulent NDV infection; however, they do not always prevent birds from shedding virulent viruses [15]. In the present study, birds that received rNDV GVII vaccine (B) groups (G6 and G7) showed remarkably higher HI antibody titers against GVII 1.1 antigen and consistently were better protected vaccinated groups. However, no significant variation was detected between the vaccinated groups after using GII LaSota antigen except for the G4 that received rNDV GVII vaccine (A), resembling the live/live model that showed the lowest values. Though live and inactivated NDV GII LaSota vaccines (G2 and G3) clinically protected the birds, they could not reduce the virulent virus shedding until the 10th DPC, and the bird’s tracheal lesion scores indicated more damage to the trachea, especially at 7 DPC. This may explain the NDV GII vaccine failures under field conditions [29,35]. In spite of significant reductions in virus shedding when used as LI/L regimens, the lower clinical protection offered by the GVII vaccine (A) was unexpected; however, histopathological findings indicated more severe deterioration of tracheal tissue in the GVII vaccine (A) vaccinated group that probably led to the observed mortalities. In general, birds vaccinated with rNDV GVII vaccines showed significantly lower virus shedding levels, considering that birds of G6 and G7 had the lowest shedding virus titers at 7 DPC. The virus shedding findings align with previous NDV vaccine studies [6,15,33,35].

Genetic engineering has offered a promising tool for developing novel vaccinations against animal and human infectious diseases. One intriguing technique is to use reverse genetics to develop an rNDV [40]. Several reports have highlighted that recombinant vaccines with a close genetic relationship with circulating viruses can provide better protection against challenges with a lethal dose of vNDV than conventional ones [11,17,41]. Several commercial rNDV vaccines have been used to immunize chickens against the disease. In our study, two commercial vaccines marketed in Egypt were used. Vaccine (A) is a chimeric recombinant GVII based on the LaSota strain backbone; however, Vaccine (B) is based on a VG/GA strain backbone. Even though cell-mediated immune response induced by the different live NDV vaccines would have offered a better opportunity for understanding the limitations of challenge virus replication and shedding, the noticeable superior performance of recombinant ND GVII vaccine (B) may be attributed to the backbone VG/GA strain. The VG/GA strain of NDV can induce a local mucosal immune response based on its characteristics as an enterotropic strain capable of replicating in the chickens’ intestinal tract efficiently [42,43].

## 5. Conclusions

In summary, the present study further highlighted the inadequate level of protection afforded by the conventional live and inactivated LaSota vaccines against the recently circulating vNDV GVII.1.1 strains. The study also emphasizes the importance of combining live and inactivated rNDV GVII 1.1 strain in vaccination programs of broiler chickens to obtain protection against clinical signs and mortalities associated with Newcastle disease. Nevertheless, the results also suggest that vaccines developed from rNDV GVII 1.1. parental VG/GA strains better reduce virus shedding in terms of the number of shedders and the amount of shed virus than those based on LaSota parent strains. By matching the vaccination to field viruses, it is possible to dramatically reduce Newcastle disease spread and hence the danger of viral evolution. Thus, it is recommended to evaluate the cell-mediated immune response induced by the novel live rNDV GVII.1.1 vaccines to understand their mechanism in better mediating the mucosal immunity against the disease.

## Figures and Tables

**Table 1:** Experimental design of vaccination schemes and post-vaccination challenge study.

Group/Number of Birds	Abbreviation ^1^	Treatment(s)	Volume/Method	Age of Birds		
				7 Days	21 Days	31 Days
**G1: (*n* = 15)** **unvaccinated, unchallenged**	**UC**	Saline	50 µL/Eye drop	x	x	-
**G2: (*n* = 15) vaccinated, challenged**	**LaSota GII L/L**	Volvac^®^ LaSota gold	50 µL/Eye drop	x	x	Velogenic NDV GVII 1.1 6.0 log_10_ EID_50_/200 µL/Eye Drop
**G3: (*n* = 15) vaccinated, challenged**	**LaSota GII LI/L**	Volvac^®^ LaSota gold	50 µL/Eye drop	x	x	
		Volvac^®^ AI + ND KV	0.5 mL/Subcutaneous	x	-	
**G4: (*n* = 15) vaccinated, challenged**	**Vaccine (A) GVII L/L**	Himmvac Dalguban N+ (Plus) (Live)	50 µL/Eye drop	x	x	
**G5: (*n* = 15) vaccinated, challenged**	**Vaccine (A) GVII LI/L**	Himmvac Dalguban N+ (Plus) (Live)	50 µL/Eye drop	x	x	
		Himmvac Dalguban N+ (Plus) (Inactivated)	0.5 mL/Subcutaneous	x	-	
**G6: (*n* = 15) vaccinated, challenged**	**Vaccine (B) GVII L/L**	RINNOVAC™ ELI-7	50 µL/Eye drop	x	x	
**G7: (*n* = 15) vaccinated, challenged**	**Vaccine (B) GVII LI/L**	RINNOVAC™ ELI-7	50 µL/Eye drop	x	x	
		MEVAC™ ND7 Plus	0.5 mL/Subcutaneous	x	-	
**G8: (*n* = 15) unvaccinated, challenged**	**CC**	Saline	50 µL/Eye drop	x	x	

^1^ G1 UC: unchallenged controls (unvaccinated), G2 LaSota GII L/L: primed with live LaSota on 7 days old then received a booster with live LaSota on 21 days old, G3 LaSota GII LI/L: primed with live and inactivated LaSota on 7 days old then received a booster with live LaSota on 21 days old, G4 vaccine (A) GVII L/L: primed with live vaccine (A) GVII on 7 days old and received a booster with live vaccine (A) GVII on 21 days old, G5 vaccine (A) GVII LI/L: primed with live and inactivated vaccine (A) GVII on 7 days old then a booster with live vaccine (A) GVII on 21 days old, G6 vaccine (B) GVII L/L: primed with live vaccine (B) GVII on 7 days old and a booster with live vaccine (B) GVII on 21 days old, G7 vaccine (B) GVII LI/L: primed with live and inactivated vaccine (B) GVII on 7 days old then received a booster with live vaccine (B) GVII on 21 days old, G8 CC: challenged controls (unvaccinated).

**Table 2:** Oligonucleotide primers and thermal profile of virulent Newcastle disease virus RT-qPCR.

Virus	Primer Sequence (5′–3′)		Reference
F	CGS-ARG-ATM-CAA-GGG-TCT		[21]
R	CTA-CAC-TGC-CAA-TAA-CRG-C		
Probe	AGG-AGA-CRA-AAA-CGY-TTT-ATA-GGT-GC		
**Thermal Profile**			
**Step**	**Temperature**	**Time**	**No. of Cycles**
cDNA synthesis	45 °C	10 min	1
Thermo-start activation	95 °C	10 min	1
Denaturation	95 °C	15 s	40
Annealing/Extension	56 °C	45 s	
Final extension	72 °C	5 min	1

**Table 3:** Mean log_2_ hemagglutination inhibition antibody titers in vaccinated birds using homologous and heterologous antigens at 21 and 31 days of age.

Group ^1^	Day 21	
	GII LaSota Antigen	GVII 1.1 Antigen
**G1: Negative control (UC)**	0.3 ± 0.0	0.5 ± 0.0
**G2: LaSota GII L/L**	^A^ 3.00 ± 1.15 ^a 2^	^A^ 3.11 ± 0.78 ^a^
**G3: LaSota GII LI/L**	^A^ 3.67 ± 1.00 ^a^	^A^ 3.40 ± 1.07 ^a^
**G4: Vaccine (A) GVII L/L**	^A^ 2.50 ± 0.55 ^a^	^A^ 2.67 ± 1.66 ^a^
**G5: Vaccine (A) GVII LI/L**	^A^ 2.60 ± 0.55 ^a^	^A^ 4.33 ± 0.50 ^b^
**G6: Vaccine (B) GVII L/L**	^A^ 3.00 ± 0.82 ^a^	^A^ 3.40 ± 1.17 ^a^
**G7: Vaccine (B) GVII LI/L**	^A^ 3.00 ± 0.89 ^a^	^A^ 4.50 ± 0.85 ^b^
**Group**	**Day 31**	
	**GII LaSota Antigen**	**GVII 1.1 Antigen**
**G1: Negative control (UC)**	0.5 ± 0.53	0.2 ± 0.42
**G2: LaSota GII L/L**	^A^ 3.80 ± 1.14 ^a^	^A^ 3.50 ± 0.93 ^a^
**G3: LaSota GII LI/L**	^A^ 4.20 ± 0.63 ^a^	^A^ 4.50 ± 0.97 ^a^
**G4: Vaccine (A) GVII L/L**	^B^ 2.89 ± 0.78 ^a^	^A^ 3.80 ± 1.03 ^a^
**G5: Vaccine (A) GVII LI/L**	^A^ 3.50 ± 0.53 ^a^	^A^ 4.50 ± 1.08 ^a^
**G6: Vaccine (B) GVII L/L**	^A^ 3.30 ± 0.67 ^a^	^A^ 4.25 ± 1.39 ^a^
**G7: Vaccine (B) GVII LI/L**	^A^ 3.60 ± 0.84 ^a^	^B^ 6.20 ± 1.03 ^b^

^1^ G1 UC: unchallenged controls (unvaccinated), G2 LaSota GII L/L: primed with live LaSota on 7 days old then received a booster with live LaSota on 21 days old, G3 LaSota GII LI/L: primed with live and inactivated LaSota on 7 days old then received a booster with live LaSota on 21 days old, G4 vaccine (A) GVII L/L: primed with live vaccine (A) GVII on 7 days old and received a booster with live vaccine (A) GVII on 21 days old, G5 vaccine (A) GVII LI/L: primed with live and inactivated vaccine (A) GVII on 7 days old then a booster with live vaccine (A) GVII on 21 days old, G6 vaccine (B) GVII L/L: primed with live vaccine (B) GVII on 7 days old and a booster with live vaccine (B) GVII on 21 days old, G7 vaccine (B) GVII LI/L: primed with live and inactivated vaccine (B) GVII on 7 days old then received a booster with live vaccine (B) GVII on 21 days old, G8 CC: challenged controls (unvaccinated). ^2^ Significance between HI antibody titers between different experimental groups is indicated by capital superscript letters on the left side; meanwhile, significant differences between titers using different diagnostic antigens in the same group are indicated by small superscript letters on the right side (*p* < 0.05).

**Table 4:** Virus shedding titers on the 3rd, 7th, and 10th days post challenge (DPC).

Groups ^1^	3 DPC		7 DPC		10 DPC	
	Mean ± SD ^2^	Positivity ^3^	Mean ± SD	Positivity	Mean ± SD	Positivity
Challenged control	5.33 ± 0.38 ^a 4^	5/5 (100%)	N/A ^5^	N/A	N/A	N/A
LaSota GII L/L	4.37 ± 0.22 ^ab^	5/5 (100%)	1.88 ± 0.20 ^a^	5/5 (100%)	1.73 ± 0.10 ^a^	5/5 (100%)
LaSota GII LI/L	3.94 ± 0.51 ^abc^	5/5 (100%)	2.31 ± 0.43 ^ab^	5/5 (100%)	1.41 ± 0.11 ^ab^	5/5 (100%)
Vaccine (A) GVII L/L	4.26 ± 0.52 ^ab^	5/5 (100%)	1.77 ± 0.46 ^ab^	4/5 (80%)	1.40 ± 0.10 ^ab^	5/5 (100%)
Vaccine (A) GVII LI/L	4.25 ± 0.46 ^ab^	5/5 (100%)	1.94 ± 0.17 ^ab^	3/5 (60%)	Nd ^6^	0/5 (0%)
Vaccine (B) GVII L/L	4.20 ± 0.30 ^ab^	5/5 (100%)	1.45 ± 0.59 ^ab^	3/5 (60%)	Nd	0/5 (0%)
Vaccine (B) GVII LI/L	3.70 ± 0.48 ^abc^	5/5 (100%)	1.13 ± 0.16 ^ab^	2/5 (40%)	Nd	0/5 (0%)

^1^ Abbreviations: GII: genotype II-based vaccine, GVII: genotype VII.1.1.-based vaccine, L/L: live vaccine at 7 and 21 days, LI/L: live and inactivated at 7 days, then live vaccine at 21 days. ^2^ The mean shedding titer ± SD is expressed as EID_50_/mL using RT-qPCR. ^3^ Positivity %: percentage of active virus shedder birds out of the tested samples. ^4^ N/A: not applicable. ^5^ Virus titers in the same column followed by different small letters are statistically different. (*p* < 0.05). ^6^ Nd: not detected.

**Table 5:** The histopathological scoring of tracheal lesions at 3 and 7 days post challenge.

Days Post Challenge	Groups ^1^	Tracheal Lesions Scores ^2^				
		Epithelial Degeneration	Epithelial Sloughing	Congestion	Lymphocytic Infiltration	Tracheal Cast
**3**	**G1: Negative control (UC)**	-	-	-	-	-
	**G2: Challenged control**	+++	++	+++	++	+
	**G2: LaSota GII L/L**	+	-	+	+	-
	**G3: LaSota GII LI/L**	+++	+	++	+	+
	**G4: Vaccine (A) GVII L/L**	++	+	+	+	+
	**G5: Vaccine (A) GVII LI/L**	+	-	+	+	-
	**G6: Vaccine (B) GVII L/L**	++	+	++	+	+
	**G7: Vaccine (B) GVII LI/L**	+	-	+	+	-
**7**	**G1: Negative control (UC)**	-	-	-	-	-
	**G2: Challenged control**	+++	++	++	+++	+++
	**G2: LaSota GII L/L**	+	-	+	+	-
	**G3: LaSota GII LI/L**	++	++	+	++	++
	**G4: Vaccine (A) GVII L/L**	++	+	+	+	-
	**G5: Vaccine (A) GVII LI/L**	+	+	+	++	-
	**G6: Vaccine (B) GVII L/L**	+	+	+	++	+
	**G7: Vaccine (B) GVII LI/L**	+	-	+	+	-

^1^ G1 UC: unchallenged controls (unvaccinated), G2 LaSota GII L/L: primed with live LaSota on 7 days old then received a booster with live LaSota on 21 days old, G3 LaSota GII LI/L: primed with live and inactivated LaSota on 7 days old then received a booster with live LaSota on 21 days old, G4 vaccine (A) GVII L/L: primed with live vaccine (A) GVII on 7 days old and received a booster with live vaccine (A) GVII on 21 days old, G5 vaccine (A) GVII LI/L: primed with live and inactivated vaccine (A) GVII on 7 days old then a booster with live vaccine (A) GVII on 21 days old, G6 vaccine (B) GVII L/L: primed with live vaccine (B) GVII on 7 days old and a booster with live vaccine (B) GVII on 21 days old, G7 vaccine (B) GVII LI/L: primed with live and inactivated vaccine (B) GVII on 7 days old then received a booster with live vaccine (B) GVII on 21 days old, G8 CC: challenged controls (unvaccinated). ^2^ Histopathological scoring of tissue injury in trachea were scored in degrees as follows: (-): no change; (+): <25% tissue damage; (++): 26–50% tissue damage; (+++): 51–75% tissue damage.

## Data Availability

Not applicable.

## Supplementary Materials

The following supporting information can be downloaded at: https://www.mdpi.com/article/10.3390/ani12131696/s1, Figure S1: Phylogenetic (A) and nucleotide & amino acid sequence identities of the F gene of vaccine and challenge ND viruses. The evolutionary history was inferred using the Neighbor-Joining method with 1000 bootstrap replicates using MEGA alignment software 11. The LaSota GII vaccine strain (●), recombinant vaccine (A) GVII strain and its backbone LaSota strain (●), recombinant vaccine (B) GVII (●) and its VG/GA backbone strain (●), and the challenge strain (●) viruses are indicated.
