# Supplementary material for: Genotype VII.1.1-Based Newcastle Disease Virus Vaccines Afford Better Protection against Field Isolates in Commercial Broiler Chickens
Source: Animals (Basel). 2022 Jun 30;12(13):1696. doi: 10.3390/ani12131696 (PMC9265022; doi:10.3390/ani12131696)
Supplement: Supplementary file 1 [file animals-12-01696-s001.zip › animals-1735833-supplementary.pdf]

A

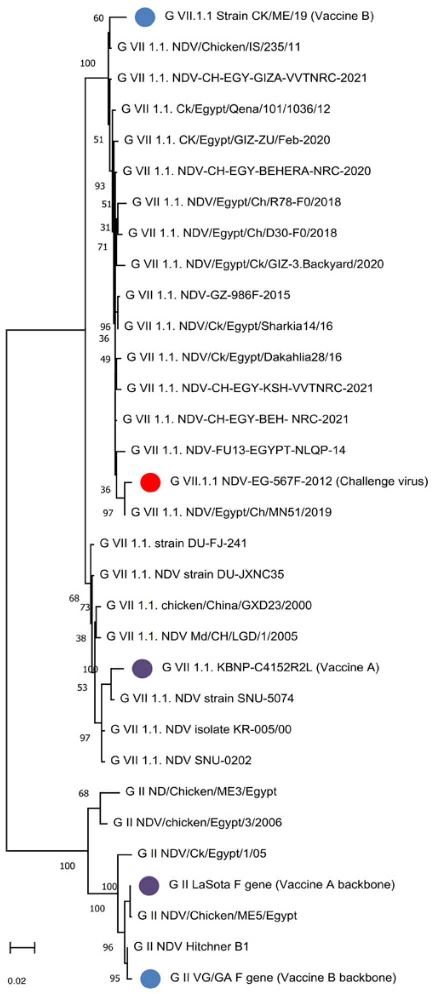

B

Virus	1	2	3	4	5	6	7	8	9	10	11	12	13	14	15	16
**1. G_VII.1.1_Strain_CK/ME/19_(Vaccine_B)** ●		97.8	97.1	97.1	97.8	97.2	97.9	94.5	95.6	96.1	96	95.5	94.5	83.4	83.4	84.2
2. G_VII_1.1._NDV-GZ-986F-2015	99		98.9	98.6	99	99.1	99.4	94.1	97.9	96.4	96.4	95.9	94.7	83.3	83.2	83.8
3. G_VII_1.1._NDV/Egypt/Ch/R78-F0/2018	98.3	98.9		98.1	98.7	98.7	98.9	93.6	97.3	95.9	95.9	95.5	94.2	83.1	83	83.8
4. G_VII_1.1._NDV/Egypt/Ch/MN51/2019	98.8	99.4	99		98.5	98.5	98.9	93.7	99.5	96	96	95.5	94.4	83.1	83	83.7
5. G_VII_1.1._CK/Egypt/GIZ-ZU/Feb-2020	98	99	99	99.2		98.7	99.2	94.4	98.8	96.8	96.6	96.2	95.1	83.5	83.4	83.9
6. G_VII_1.1._NDV/Ck/Egypt/Dakahlia28/16	98.6	99.4	99	99.6	99.1		99.3	94	98.3	96.3	96.3	95.8	94.6	83.3	83.3	83.9
7. G_VII_1.1._NDV-CH-EGY-BEH-_NRC-2021	98.7	99.6	99.2	99.8	99.1	99.6		94.6	98.5	97	96.9	96.4	95.3	83.7	83.6	84.3
**8. G_VII_1.1._KBNP-C4152R2L (Vaccine_A)** ●	96.4	96.4	96	96.5	96	96.6	96.5		91.5	97.5	97.4	97.5	98.9	84.4	84.4	85.1
**9. GVII_EG_567F_2012 (Challenge Virus)** ●	94.9	98.4	98.4	99.2	97.8	97.8	97.1	91.2		94.4	94.7	93.7	93.2	79.6	79.4	80.8
10. G_VII_1.1._NDV_strain_DU-JXNC35	97.5	98.3	97.9	98.5	98	98.6	98.5	98	95.6		99.3	99.5	98.2	84.8	84.8	85.6
11. G_VII_1.1._strain_DU-FJ-241	97.1	97.9	97.5	98.1	97.6	98.2	98.2	97.8	94.9	99.5		99.4	98	84.8	84.8	85.6
12. G_VII_1.1._NDV_Md/CH/LGD/1/2005	96.6	97.3	97	97.5	97.1	97.6	97.6	97.5	94.2	99.1	99.3		98.1	84.7	84.8	85.6
13. G_VII_1.1._NDV_strain_SNU-5074	96.2	97	96.6	97.1	96.7	97.3	97.3	98.6	92.7	98.7	98.6	98.2		84.8	84.8	85.6
**14. G_II_LaSota _(Vaccine_A_backbone)** ●	88.4	89	88.6	88.9	88.2	88.2	88.5	89	80.3	89	89	88.4	89		98.9	95.7
**15. G_II_VG/GA _(Vaccine_B_backbone)** ●	88.1	88.6	88.2	88.5	87.9	87.9	88.2	88.6	79.6	88.6	88.6	88.1	88.6	99.3		95.5
16. G_II_ND/Chicken/ME3/Egypt translation	89.7	89.9	89.1	89.8	88.8	89.3	89.6	89.5	81.8	90.1	90.1	89.5	89.5	95.1	94.4	

Amino acid identities (%)

Nucleotide identities (%)

Supplementary Figure S1. Phylogenetic (A) and nucleotide & amino acid sequence identities of the F gene of vaccine and challenge ND viruses. The evolutionary history was inferred using the Neighbor-Joining method with 1000 bootstrap replicates using MEGA alignment software 11. The LaSota GII vaccine strain (●), recombinant vaccine (A) GVII strain and its backbone LaSota strain (●), recombinant vaccine (B) GVII (●) and its VG/GA backbone strain (●), and the challenge strain (●) viruses are indicated
